# Predicting delirium in acute ischemic stroke: the PREDELIS score

**DOI:** 10.1007/s00415-025-13073-5

**Published:** 2025-05-11

**Authors:** Natalie Berger, Diether Kramer, Michael Schrempf, Edith Hofer, Alexander Pichler, Simon Fandler-Höfler, Melanie Haidegger, Isra Hatab, Martin Heine, Jan Jagiello, Herbert Koller, Stefan Lilek, Sai Veeranki, Christian Enzinger, Thomas Gattringer, Markus Kneihsl

**Affiliations:** 1 Department of Neurology, Medical University of Graz, Auenbruggerplatz 22, 8036 Graz, Austria; 2 Directorate Technology and IT, Steiermärkische Krankenanstaltengesellschaft M.B.H., Graz, Austria; 3 Division of Cardiolody, Department of Internal Medicine, Medical University of Graz, Graz, Austria; 4 Institute for Medical Informatics, Statistics and Documentation, Medical University of Graz, Graz, Austria; 5 Department of Neurology, LKH Oststeiermark, Feldbach, Austria; 6 Department of Neurology, LKH Murtal, Knittelfeld, Austria; 7 Department of Neurology, LKH Graz II, Graz, Austria; 8 Department of Neurology, LKH Hochsteiermark, Bruck/Mur, Austria; 9 Division of Neuroradiology, Department of Radiology, Medical University of Graz, Graz, Austria

**Keywords:** Delirium, Acute ischemic stroke, Prediction score, Prevention

## Abstract

**Background:**

Delirium, defined as an acute, fluctuating disturbance in consciousness, attention and cognition, is a common stroke complication and associated with poor functional outcome. Although resource-intensive prevention strategies could reduce delirium rates, their implementation in unselected stroke patients is challenging. This study aimed to develop a risk score for predicting delirium in acute ischemic stroke (PREDELIS).

**Methods:**

We retrospectively included all ischemic stroke patients admitted to five stroke units of Styria, Austria, between 2013 and 2021. Data were retrieved from a comprehensive medical information system using semi-automated data extraction. The PREDELIS score was based on multivariable logistic regression analysis to identify admission variables associated with delirium.

**Results:**

14,475 acute ischemic stroke patients (median age: 76 years, 46% women) were split in a 40% derivation (n = 6151; delirium = 398, 6.5%) and a 60% validation cohort (n = 8324; delirium: 568, 6.8%). Previous delirium (4 points), chronic alcohol consumption (3), age > 70 years (2), male sex (2), infection (2), admission NIHSS > 7 (1), non-lacunar stroke (1) and vision/hearing impairment (1) were associated with delirium (all p < 0.05) and included in our score (median: 5 points). The score´s area under the curve was 0.72 in both the derivation (95% CI 0.69–0.75) and the validation cohort (95% CI 0.70–0.74). While patients with a score of ≤ 5 had a low delirium risk (2.5%), a score of ≥ 9 indicated a high risk (30.9%).

**Discussion and conclusion:**

This study introduces a novel score for early delirium risk estimation in ischemic stroke patients, aiding clinicians in identifying high-risk individuals for targeted screening and prevention.

**Supplementary Information:**

The online version contains supplementary material available at 10.1007/s00415-025-13073-5.

## Introduction

Delirium is a common complication in acute ischemic stroke, and characterized by acute disturbances in consciousness, attention, and cognition, typically exhibiting a fluctuating course. The diagnosis of delirium is generally made using either the Diagnostic and Statistical Manual of Mental Disorders, Fifth Edition (DSM- 5) or the International Classification of Diseases, Tenth Revision (ICD- 10) diagnostic criteria [1, 2]. Both diagnostic systems distinguish delirium from mimicking conditions, such as depression, anxiety or dementia, by emphasizing its acute onset, fluctuating course, and disorganized thinking. The treatment of delirium involves, (a) addressing modifiable individual risk factors, (b) enhancing non-pharmacological interventions, and, when necessary to protect the patient and/or personnel, (c) initiating specific pharmacological therapy.

In patients with acute ischemic stroke delirium is associated with a prolonged hospital stay, higher readmission rates, poor functional outcome and mortality [3–6].

Therefore, delirium prevention may be crucial for improving post-stroke prognosis.

In hospitalized non-stroke cohorts, non-pharmacological multicomponent strategies have been shown to reduce delirium rates by more than 30% [7, 8]. These strategies include orientation support through clocks, calendars, and verbal reorientation, as well as addressing sensory impairments by ensuring access to glasses and hearing aids. Sleep enhancement involves minimizing nighttime noise and light to maintain regular sleep–wake cycles. Early mobilization—through sitting up, walking, and movement exercises—combined with adequate hydration and nutrition is essential for reducing delirium risk. However, implementation in unselected stroke patients is challenging due to time constraints, staff availability, and financial resource. Early identification of patients at high risk for delirium could significantly aid in directing therapeutic efforts towards individuals who are likely to benefit from intensified interventions [9]. Although validated delirium risk scores exist for elderly hospitalized non-stroke patients (i.e., Delirium risk assessment score [DRAS]), data on delirium prediction in stroke patients are scarce [5, 9–12]. The few available studies reporting on delirium risk in acute stroke were all constrained by small numbers of patients with delirium, heterogeneous treatment settings (e.g., intensive care unit versus stroke unit), and were not specifically developed for ischemic stroke patients [9–11].

This prompted us to use a large and homogenous cohort of ischemic stroke patients uniformly treated at a stroke unit to develop a scoring instrument for Predicting Delirium in acute Ischemic Stroke patients (PREDELIS). Additionally, we aimed to test the performance of our model against previously published scores in a validation cohort.

## Methods

The data of our study are available from the corresponding author upon reasonable request. The study was approved by the local ethics committee (30–146 ex 17/18). Reporting was performed according to the Strengthening the Reporting of Observational Studies in Epidemiology (STROBE) statement.

### Study setting and data acquisition

In this retrospective multicenter study, we included all acute ischemic stroke patients that had been admitted to one of the five stroke units in the federal state of Styria (Austria) between 2013 and 2022. These stroke units collectively provide stroke care for over 1.2 million inhabitants [13]. Ischemic stroke was defined according to imaging-based criteria, utilizing either magnetic resonance imaging (MRI) or computed tomography (CT) scans. Patients less than 18 years of age were excluded from the study as were patients who were comatose or had died on the day of admission (Supplemental Fig. 2) [4]. Moreover, patients on mechanical ventilation were not included in the study, as they were routinely treated at a neurointensive care unit.

All study related data was extracted from the hospital information system openMEDOCS [14]. The system encompasses all medical information documented in every public hospital within the province of Styria, including data from medical reports, nursing documentation, and laboratory and diagnostic imaging reports. Additionally, the Austrian Stroke Unit Registry [15], which contains stroke-specific data prospectively recorded by the attending vascular neurologist, is integrated within openMEDOCS. For data retrieval, semi-automated data extraction tools including natural language search (NLS) were employed by two experts in big data analytics (DK, MS).

### Study variables

We extracted data on demographics, medical history, comorbidities, medication, vascular risk factors and stroke-specific information. This included variables that have previously been reported to be associated with delirium in stroke patients, namely age, male sex, infarct location (anterior/posterior circulation stroke, non-lacunar stroke, affected hemisphere, stroke severity using the National Institutes of Health Stroke Scale (NIHSS) score, pre-existing mobility problems according to a modified Rankin Scale (mRS) score of ≥ 3, stroke symptoms (i.e., aphasia, neglect), infections, atrial fibrillation, previous delirium (documented episode of delirium during a prior hospital stay), chronic alcohol consumption, polypharmacy (defined as ≥ 5 drugs; outpatient medication), multimorbidity (defined as ≥ 2 chronic diseases), and dementia/cognitive impairment (Table 1) [6, 9–11, 16–21]. Pre-existing dementia/cognitive impairment were defined according to the international classification of diseases (ICD)− 10 diagnosis as documented in the medical records (i.e., F00-F02 or U51.11–12), or if patients received respective medications [1]. Chronic alcohol consumption was defined as consuming ≥ 2 drinks or ≥ 20 g of ethanol on at least 5 days per week [22].

**Table 1:** Variables analyzed in the present study

Categories	Variables	
Demographics	Age*	Male sex*
Vascular risk factors	Arterial hypertension* Hyperlipidemia Diabetes*	Smoking* Chronic alcohol consumption * Artrial fibrillation*
Laboratory parameters	Leucocytes* Platelets Hematocrit Blood urea* MCV Creatinine* Blood sodium* Blood potassium*	Bilirubine GGT Aspartate aminotransferase Alanine aminotransferase Lipase Glucose* CRP
Medical history	Dementia/mild cognitive impairment* Previous stroke* Previous delirium* Drug abuse Renal insufficiency* Chronic liver disease Psychiatric disease* Previous heart disease*	Malnutrition* Infection* Visual/hearing impairment* Epilepsy* Multimorbidity (≥ 2 chronic diseases)* Mobility problems at admission*
Medication	Sedative medication at admission*	Polypharmacy (≥ 5 drugs at admission)*
Stroke specific factors	Anterior circulation stroke Posterior circulation stroke Non-lacunar stroke Infratentorial stroke Supratentorial stroke Left/right hemispheric stroke	NIHSS at admission Intravenous thrombolysis Aphasia Neglect Dysphagia*

Marked variables (*) were associated with delirium in non-stroke cohorts; underlined variables were associated with delirium in stroke cohorts in previous studies.

*CRP* c-reactive protein, *GGT* gamma-glytamyl transferase, *MCV* mean corpuscular volume, *mRS* modified Rankin scale, *NIHSS* National Institutes of Health Stroke scale

Moreover, we collected factors associated with delirium in hospitalized non-stroke patients that have not yet been investigated in stroke cohorts (i.e., anticholinergic medication, malnutrition, and electrolyte imbalances) [4, 12, 23, 24]. For laboratory variables, we always used data from laboratory assessments at hospital admission. All documented variables are presented in Table 1 and Supplemental Table 1.

### Delirium

Delirium was diagnosed based on the ICD- 10 diagnostic code (F05) documented during the hospital stay or in the Austrian Stroke Unit Registry [1, 2, 15]. No uniform delirium screening tool was used, but all included patients were continuously monitored by nurses and neurologists specialized in stroke care and aware of delirious states. To avoid an underestimation of delirium, NLS was used to search unstructured text for key words indicating delirious states: “delirium”, “delirious state”, “hyperactive”, “hypoactive”, “apathic”, “agitation”, “confusion”, “hallucination”, “restlessness”, “unsettled” and “anxious” [25–28]. If key words were identified in patients without a diagnosis of delirium, patient’s medical history was manually reviewed retrospectively by stroke experts (MK, TG). Again, delirium was diagnosed if the respective ICD- 10 diagnostic criteria were fulfilled [1, 2].

### Statistical analyses

Statistical analysis was performed using IBM SPSS Statistics, version 29, MEDCAL and using R Statistical Programming Language Version 3.6.2.

Score development: For developing the PREDELIS score, the total cohort was randomly split into two groups to form a derivation and validation cohort (Supplemental Table 2). We allocated 40% of the cohort to the derivation set and 60% to the validation set, aiming to achieve robust validation results with more precise estimates of predictive accuracy, enhanced generalizability, and reduced risk of overfitting [29].

In the next step, we performed a univariable analysis to assess the association of all documented variables with delirium in acute stroke patients. Pearson’s chi-square or Fisher’s exact test was used to compare dichotomous variables. All quantitative variables were first tested for Gaussian distribution with the Kolmogorov–Smirnov test and, if Gaussian distribution was identified, a two-sample independent t-test was utilized to compare the variables. The Mann–Whitney-U-Test was used for non-parametric data.

After correction for multiple testing using the Bonferroni method, only variables with p-values < 0.001 were considered statistically significant, namely age, male sex, NIHSS at admission, atrial fibrillation, non-lacunar stroke, dementia/cognitive impairment, previous delirium, chronic alcohol consumption, visual/hearing impairment, infection at admission, abnormal hematocrit and dysphagia. To assess multicollinearity, we conducted formal collinearity diagnostics (Supplemental Table 3). Notably, C-reactive protein was excluded from the multivariable analysis due to its strong correlation with infection with a Phi coefficient > 0.7 and a variance inflation factor > 10. The remaining 12 variables were included in a logistic regression model using the stepwise forward selection method. We used β coefficients from the logistic regression model to develop the PREDELIS score.

Score performance, validation and calibration: To assess the performance of the PREDELIS score we calculated the area under the receiver-operating characteristics curve (AUC). We also obtained sensitivity, specificity and accuracy measures for score cutoffs to allow to judge on its potential clinical utility. Cutoffs were established based on a previously proposed delirium risk stratification in acute stroke patients by Oldenbeuving et al. [11], defining low delirium risk as ≤ 5%, moderate delirium risk as > 5% to < 20%, and high delirium risk as ≥ 20% [11].

The score was validated internally in the randomly selected 60% validation cohort. A calibration plot was used to display the relationship between predicted probabilities of delirium development and observed probabilities in the validation cohort (Supplemental Fig. 3).

Moreover, we compared the score with delirium prediction models that have previously demonstrated a high accuracy for predicting delirium in stroke (Oldenbeuving et al.; Model 1 and 2) and non-stroke cohorts (DRAS) [11, 12]. To statistically compare AUC differences between PREDILIS, Oldenbeuving Model 1 and 2 and the DRAS, we applied DeLong`s test to all models. Details on the diagnostic test evaluation of the scores are presented in Supplemental Table 4.

## Results

Between January 2013 and December 2021, a total of 19,954 patients with suspected stroke were admitted to a Stroke Unit in the federal state of Styria. We excluded patients who did not have a final diagnosis of ischemic stroke (n = 5353) and those who were in coma at admission (n = 78) or had died within 24 h of admission (n = 48), resulting in a final study cohort of 14,475 patients (median age: 76 years, interquartile range (IQR): 66–83 years; 46% female). Baseline parameters are displayed in Tables 2 and 3, and Supplemental Table 5.

**Table 2:** Demographics and medical history of all patients included in the study, categorized by the presence of delirium

Variables	All ischemic stroke patients (n = 14,475)	Derivation cohort			
		Delirium (n = 398)	No delirium (n = 5753)	OR (95% CI)	p-value*
Demographics					
Age in years, median (IQR)	76 (66–83)	78 (70–84)	75 (65–83)		**< 0.001**
Age > 70 years, n (%)	9737 (67.3)	300 (75.0)	3792 (65.9)	1.58 (1.25–2.00)	**< 0.001**
Male sex, n (%)	7802 (53.9)	267 (67.0)	2987 (51.9)	1.89 (1.52–2.34)	**< 0.001**
Medical history, n (%)					
Atrial fibrillation	4297 (29.7)	156 (39.2)	1628 (28.3)	1.63 (1.33–2.01)	**< 0.001**
Arterial hypertension	12,194 (84.2)	345 (86.7)	4832 (84.0)	1.24 (0.92–1.67)	0.177
Diabetes	3732 (25.8)	98 (24.6)	1432 (24.9)	0.99 (0.78–1.25)	0.952
Hyperlipidemia	3017 (49.0)	205 (51.5)	2812 (48.9)	1.11 (0.91–1.36)	0.325
Smoking	2254 (17.6)	88 (22.1)	1048 (18.2)	1.27 (1.00–1.63)	0.061
Chronic alcohol consumption	1458 (10.1)	84 (21.1)	549 (9.5)	2.54 (1.96–3.28)	**< 0.001**
Dementia/mild cognitive impairment	1067 (7.4)	49 (12.3)	408 (7.1)	1.84 (1.34–2.52)	**< 0.001**
Previous delirium	186 (1.3)	27 (6.7)	53 (0.9)	7.83 (4.88–12.59)	**< 0.001**
Drug abuse	1245 (8.6)	48 (12.1)	471 (8.2)	1.54 (1.12–2.11)	0.011
Renal insufficiency	2194 (15.2)	82 (20.6)	828 (14.4)	1.54 (1.20–1.99)	0.001
Chronic liver disease	5646 (39.0)	180 (45.2)	2201 (38.3)	1.33 (1.09–1.64)	0.007
Previous heart disease	9079 (62.7)	268 (67.3)	3485 (60.6)	1.34 (1.08–1.67)	0.008
Psychiatric disease	2891 (20.0)	78 (19.6)	1081 (18.8)	1.05 (0.82–1.36)	0.691
Epilepsy	778 (12.6)	55 (13.8)	723 (12.6)	1.11 (0.83–1.50)	0.483
Malnutrition	250 (1.7)	8 (2.0)	85 (1.5)	1.37 (0.66–2.84)	0.391
Visual/hearing impairment	5036 (34.8)	179 (45.0)	1899 (33.1)	1.66 (1.35–2.04)	**< 0.001**
Multimorbidity (≥ 2 chronic diseases)	10,163 (70.2)	295 (74.0)	3956 (68.8)	1.30 (1.03–1.64)	0.025

* p-values that remained significant after Bonferroni correction are highlighted in bold

*CI* confidence interval, *OR* odds ratio, *IQR* interquartile range

**Table 3:** Clinical and laboratory characteristics on admission of all patients included in the study, categorized by the presence of delirium

Variables	All ischemic stroke patients (n = 14,475)	Derivation cohort			
		Delirium (n = 398)	No delirium (n = 5753)	OR (95% CI)	p-value*
Clinical parameters, n (%)					
Infection	6061 (41.9)	248 (62.3)	2308 (40.1)	2.47 (2.00–3.04)	**< 0.001**
Pre admission mRS ≥ 3	1977 (13.7)	62 (15.6)	336 (5.8)	1.18 (0.89–1.57)	0.257
NIHSS > 7 on admission	4101 (28.3)	163 (41.0)	1565 (27.2)	1.86 (1.51–2.29)	**< 0.001**
Intravenous thrombolysis	1475 (24.0)	97 (24.4)	1378 (24.0)	1.02 (0.81–1.3)	0.856
Aphasia	3641 (25.2)	117 (29.4)	1394 (24.2)	1.31 (1.04–1.63)	0.022
Neglect	1747 (12.1)	64 (16.1)	649 (11.3)	1.51 (1.14–1.99)	0.006
Dysphagia	1350 (9.3)	60 (15.1)	487 (8.5)	1.92 (1.44–2.57)	**< 0.001**
Stroke syndrome, n (%)					
Anterior circulation stroke	7909 (54.6)	240 (60.3)	3074 (53.4)	1.32 (1.08–1.63)	0.008
Posterior circulation stroke	2485 (17.2)	79 (19.9)	987 (17.2)	1.20 (0.93–1.54)	0.171
Non-lacunar stroke	10,581 (73.1)	322 (89.9)	4142 (72.0)	1.65 (1.28–2.13)	**< 0.001**
Supratentorial stroke	5078 (82.6)	336 (84.4)	4742 (82.4)	1.16 (0.87–1.53)	0.339
Infratentorial stroke	1060 (17.2)	62 (15.6)	998 (17.3)	0.88 (0.67–1.16)	0.410
Left hemispheric stroke	6499 (44.9)	181 (45.5)	2617 (45.5)	1.00 (0.82–1.23)	1
Right hemispheric stroke	5257 (36.3)	149 (37.4)	2059 (35.8)	1.07 (0.87–1.32)	0.517
Laboratory variables					
CRP (> 5 mg/L)	5609 (38.7)	207 (52.0)	2148 (37.3)	1.82 (1.48–2.23)	**< 0.001**
Leucozytes (> 11.3 * 10^9/L)	3120 (21.6)	97 (24.3)	1222 (21.2)	1.20 (0.94–1.52)	0.146
Hematocrit (> 45%)	5147 (35.6)	181 (45.5)	1952 (33.9)	1.62 (1.32–1.99)	**< 0.001**
Creatinine (> 1.2 mg/dL)	4319 (29.8)	136 (34.2)	1694 (29.4)	1.24 (1.00–1.54)	0.047
GGT (> 55 U/L)	3635 (25.1)	121 (30.4)	1398 (24.3)	1.36 (1.09–1.70)	0.008
Medication on admission					
Polypharmacy (≥ 5 drugs)	3545 (24.5)	101 (25.4)	1334 (23.2)	1.13 (0.89–1.42)	0.327
Sedative medication	314 (2.2)	14 (3.5)	111 (1.9)	1.85 (1.05–3.26)	0.041

* p-values that remained significant after Bonferroni correction are highlighted in bold

*CRP* c-reactive protein, *GGT* gamma-glytamyl transferase; *mRS* modified Rankin scale, *NIHSS* National Institutes of Health Stroke scale

Patients were randomly divided into a score derivation cohort (n = 6151; 40%) and a validation cohort (n = 8324; 60%). Clinical characteristics were comparable in both cohorts (Supplemental Table 2).

### PREDELIS score—derivation cohort

In the score derivation cohort (median age: 76 years, IQR: 70–84 years), delirium was diagnosed in 398 patients (6.5%). Patients that developed delirium were older (78 versus 75 years, p < 0.001) and more often male (67.0% versus 51.9%, p < 0.001) (Tables 2 and 3). Apart from age and sex, multivariable regression analysis depicted a previous delirium (odds ratio (OR) 6.3, 95% confidence intervals (CI) 3.8–10.4), chronic alcohol consumption (OR 2.3, 95% CI 1.8–3.1), infection at admission (OR 2.1, 95% CI 1.7–2.7) and visual/hearing impairment (OR 1.5, 95% CI 1.2–1.8) as the most important predictors for delirium in acute ischemic stroke patients. Additionally, non-lacunar stroke etiology (OR 1.4, 95% CI 1.1–1.8) and an NIHSS score > 7 at admission (OR 1.4, 95% CI 1.1–1.8) were identified as stroke-specific delirium predictors (Table 4).

**Table 4:** Multivariate logistic regression model including variables associated with delirium and score points of the PREDELIS score

Variable	ß coefficient	SE	OR	95% CI	p-value	Points
Previous delirium	1.84	0.26	6.28	3.78–10.43	< 0.001	4
Chronic alcohol consumption	0.84	0.14	2.33	1.76–3.08	< 0.001	3
Infection	0.76	0.11	2.14	1.72–2.68	< 0.001	2
Male sex	0.74	0.12	2.09	1.66–2.64	< 0.001	2
Age (> 70)	0.47	0.13	1.60	1.23–2.07	< 0.001	2
Vision/hearing impairment	0.39	0.11	1.46	1.17–1.82	< 0.001	1
Non-lacunar stroke	0.34	0.14	1.40	1.07–1.84	0.016	1
NIHSS > 7 on admission	0.33	0.12	1.39	1.10–1.76	0.005	1

*CI* confidence interval, *NIHSS* National Institutes of Health Stroke Scale, *OR* odds ratio, *PREDELIS* Predicting delirium in acute ischemic stroke, *SE* standard error

The median score of the delirium prediction model was 5 points (IQR: 3–6) yielding an AUC of 0.72 (95% CI 0.69–0.75) for the presence of delirium in acute ischemic stroke patients.

For each one-point increase in the risk score, the absolute risk of delirium increased by an average of 2.9%. Based on the predefined risk stratification, low delirium risk (≤ 5%) was observed at a cutoff of ≤ 5 points (n = 3764; 61.2% of total cohort), while the high-risk cutoff (delirium risk: ≥ 20%) was identified at ≥ 9 score points (n = 387, 6.3%). 2000 patients (32.5%) had a moderate risk (> 5% and < 20%) for delirium.

Patients with a score of ≥ 9 (delirium: 88 of 387 patients, 22.7%) had a more than sevenfold increased risk (risk ratio: 7.2; 95% CI 5.6–9.3) of developing delirium compared to those with a score of ≤ 5 (delirium: 119 of 3764 patients, 3.2%). Admission scores of ≥ 9 had a 95.2% specificity for delirium in acute ischemic stroke, while a score of ≤ 5 had a 70.1% specificity for predicting the absence of delirium. Accuracy parameters for different cutoff values are provided in the Supplemental Table 6.

### PREDELIS score—validation cohort

In the validation cohort, 568 acute ischemic stroke patients (6.8%) were diagnosed with delirium during hospital stay (median age: 76 years, IQR: 69–86 years; male sex: 45.3%).

Results in the validation cohort were similar to those in the derivation cohort, with an AUC of 0.72 (95% CI 0.70–0.74) for predicting delirium in acute ischemic stroke. For each additional point in the risk score, the absolute delirium risk increased by 3.3%. Again, patients with a score of ≤ 5 (n = 4973, 59.2% of total cohort) had a low delirium risk of 2.5%, while those with a score of ≥ 9 (n = 132; 8.9% of the total cohort) had a delirium risk of 30.9%.

The risk of delirium increased 12-fold (risk ratio = 12.3; 95% CI 9.1–14.9) when comparing score points of ≤ 5 and ≥ 9. A cutoff of ≥ 9 points demonstrated a high specificity (94.4%) for delirium in acute stroke patients, whereas a score of ≤ 5 had a specificity of 70.0% for predicting the absence of delirium in acute ischemic stroke patients.

Of note, the calibration plot shows a good agreement between the predicted and the observed delirium risk in the validation cohort (Supplemental Fig. 3).

### Risk score comparison

Low AUCs were observed when applying previously reported delirium prediction models to the validation sample, resulting in an AUC of 0.65 (95% CI 0.63–0.67) for both models of Oldenbeuving et al. and an AUC of 0.64 (95% CI 0.62–0.66) for the DRAS score. When performing DeLong’s test for statistical comparison, the PREDELIS score demonstrated a significantly higher predictive value for delirium (p < 0.001) compared to all tested scores. Notably, the other scores (DRAS, Oldenbeuving Model 1 and 2) showed no significant differences among each other (p > 0.1).

**Fig. 1:**
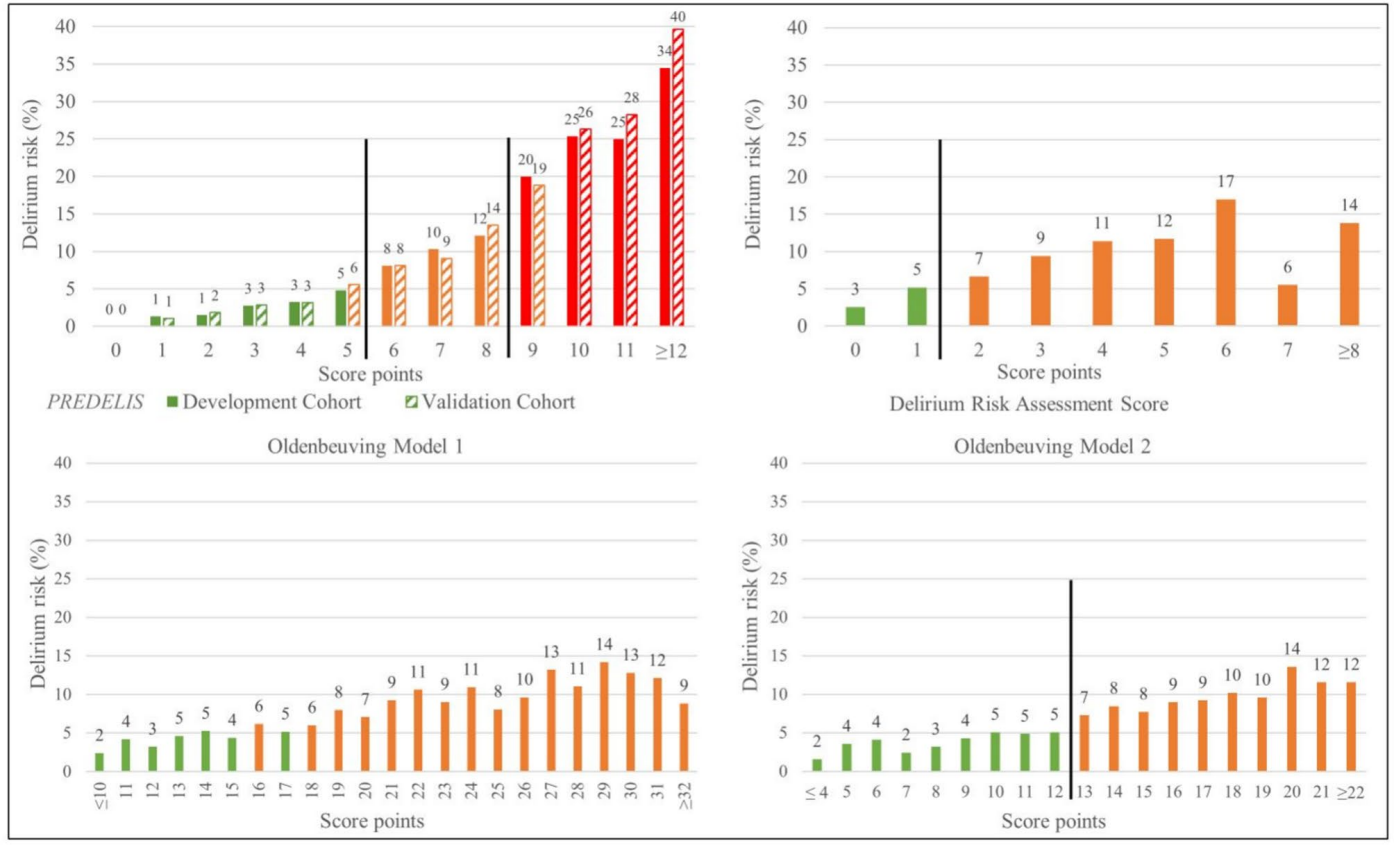
Delirium risk according to the PREDELIS score, the Delirium Risk Assessment Score (DRAS) and Oldenbeuving et al. (Model 1 and Model 2)

**Fig. 2:**
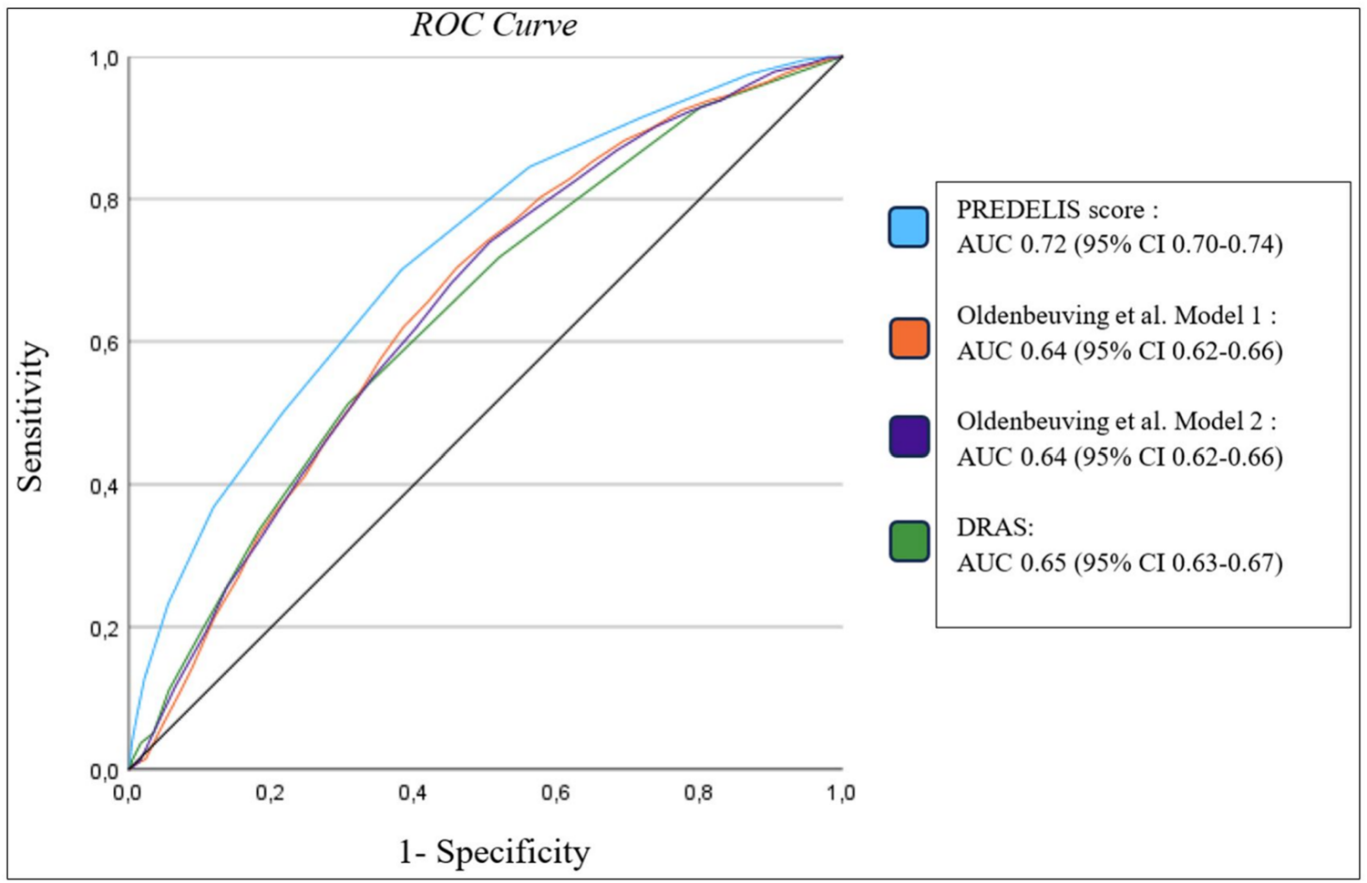
Comparison of the receiver operating characteristics curves of different scores for estimating delirium risk in acute stroke patients

The low-risk cutoff (delirium risk ≤ 5%) identified 3700 (44.4%) patients based on Oldenbeuving Model 1, 3984 (47.8%) patients based on Oldenbeuving Model 2, and 3890 (46.7%) patients based on the DRAS model, which was lower compared to the percentage of low-risk patients based on our score (n = 4973, 59.2%, p < 0.001). Notably, Oldenbeuving Models 1 and 2, as well as the DRAS, did not identify any patients with a high risk (≥ 20%) for developing delirium in acute stroke, while our score identified 427 patients (8.9%) at high risk. Details on score comparison and accuracy parameters for different cutoffs are presented in Figs. 1 and 2, and Supplemental Table 6.

## Discussion

This study developed and validated a clinical risk score (PREDELIS) that reliably predicts the risk for delirium in acute ischemic stroke patients with variables available at admission. Moreover, we compared our model with previously published scores.

Delirium prediction could be crucial for identifying high-risk patients who would benefit from resource-intensive prevention strategies. However, all previous studies on predicting delirium in acute stroke patients were limited by (very) small cohorts (largest cohort of patients with delirium: n = 62) [9–11, 30, 31].

In contrast, we had data on more than 14,000 acute ischemic stroke patients with 966 patients with delirium available. Based on the large number of included patients, we were able to internally validate the PREDELIS score and identified a robust score performance showing identical AUCs (i.e., 0.72) in both the derivation and the validation cohort. Of note, previously proposed scores that were deemed valuable for delirium prediction in stroke and non-stroke cohorts, showed a poor performance in our study cohort (AUC: all ≤ 0.65) [11, 12]. Moreover, none of these scores were able to identify high-risk patients (≥ 20% risk) for developing delirium [11, 12], while our proposed model detected over 400 patients (9% of the total cohort) that were at high risk for developing delirium. These individuals might therefore particularly benefit from delirium prevention bundles.

Vice versa, our score identified a large subgroup of ischemic stroke patients (59% of the total cohort) with a very low risk of ≤ 5% for developing delirium during hospital stay. Again, the number of patients was higher compared to previously proposed delirium prediction scores (44–48% of total cohort) [11, 12], which indicates a reliable stratification for the clinically important high- and low-risk groups. Delirium prevention strategies might therefore be less effective in these patients, allowing staff resources to concentrate on high- and intermediate-risk patients for targeted prevention efforts [31].

In this context, our study approach of using big data analytics including NLS for medical, nursing, laboratory and imaging data, allowed a comprehensive overview on individual delirium risk factors in acute stroke patients. Apart from well-known non-modifiable risk factors (i.e., age and sex) [24], we identified conditions, that might need specific prevention strategies. In our study, chronic alcohol consumption was a strong risk factor for delirium in acute stroke patients and was prevalent in barely every fourth patient with delirium. This emphasizes the relevance of assessing alcohol consumption at every admission as such patients need thorough observation and might benefit from specific (early) medical treatment (i.e., benzodiazepines, alpha2-agonists) [32]. Second, we identified clinical or laboratory signs of infection at admission as important delirium predictors. Although the recently published PRECIOUS trial [33] could not identify an outcome benefit of treating unselected acute stroke patients with ceftriaxone and paracetamol, early use of antipyretic agents and – if necessary—antibiotics could possibly be targeted in future intervention studies to reduce delirium rates or severity.

Our data strongly support previous studies in non-stroke cohorts indicating that visual/hearing impairment serves as important risk factor for delirium development [12, 24, 32]. In this context, the use of glasses and hearing aids were key elements of intervention bundles to reduce delirium in elderly hospitalized patients [5]. For acute ischemic stroke patients, the present study underlines such an approach as promising and easily applicable strategy to reduce delirium risk.

While the presence and influence of stroke-specific risk factors (i.e., stroke severity, non-lacunar stroke etiology) on delirium development was comparable to those of previous studies [9, 11, 16, 17, 33], it is important to note that although dementia/cognitive impairment was strongly associated with delirium in univariable analysis, its predictive capacity was insufficient for inclusion in our delirium prediction score. This might be attributed to the significant correlation between dementia and factors that were strongly associated with delirium in acute stroke, namely age, previous delirium and visual/hearing impairment (all p < 0.001, data not shown) [32, 34].

The present study has some limitations: First, the retrospective design did not allow for analysis of the time interval between admission and delirium diagnosis nor for assessment of prodelirogenic factors over the course of hospitalization including the administration of sedative drugs. Given the well-established association between sedative drug administration and delirium onset, this information could have been useful for applying a dynamic score that is recalculated daily after admission to account for changing parameters and should be included in future studies.

However, it is well-known that delirium typically occurs within the first 48–72 h after the event, supporting the relevance of our delirium prediction model, which is based on parameters available at admission. Moreover, prevention strategies should be applied as early as possible to reduce the incidence and duration of delirium in hospitalized patients [7].

Second, hemiparesis, aphasia, and vision or hearing impairment may have limited the accurate assessment of delirium, potentially leading to both underdiagnoses or false positive delirium diagnoses. In our study the diagnosis of delirium was based on diagnostic codes and clinical records and we cannot exclude that we might have missed some cases. Nevertheless, we employed an additional NLS-based search for key terms related to delirium across all medical documentation and manually confirmed the results. Therefore, we are confident that we did not miss a relevant number of cases, and any potential omissions likely involved patients with less-intense delirium.

Another limitation is that we only had internal validation available to assess the performance of the PREDELIS score, which may overestimate its accuracy compared to external scores (e.g., DRAS, Oldenbeuving Model 1 and 2). While these previously proposed scores showed limited predictive performance for delirium in acute ischemic stroke patients within our cohort, further external validation is necessary to confirm whether PREDELIS offers a more reliable alternative. However, our results highlight the need for a targeted approach to delirium prediction in acute ischemic stroke patients and underscore the importance of further research and validation studies in this field.

In conclusion, this study presents a new score for the early estimation of delirium risk in acute ischemic stroke patients. The score could help clinicians to preselect high-risk patients for targeted delirium screening and prevention strategies. Moreover, the presented risk factors could serve to develop stroke-specific delirium prevention bundles.

## Supplementary Information

Below is the link to the electronic supplementary material.

Supplementary file1 (DOCX 450 KB)

## Data Availability

Study data that support the findings of this study are available from the corresponding author upon reasonable request.
