# Supplementary material for: Predicting delirium in acute ischemic stroke: the PREDELIS score
Source: J Neurol. 2025 May 11;272(6):391. doi: 10.1007/s00415-025-13073-5 (PMC12066373; doi:10.1007/s00415-025-13073-5)
Supplement: Supplementary file 1 — Supplementary file1 (DOCX 450 KB) [file 415_2025_13073_MOESM1_ESM.docx]

**Supplemental Material**

**Predicting Delirium In Acute Ischemic Stroke: The PREDELIS Score**

Natalie Berger (MD)^1^, Diether Kramer (PhD)^2^, Michael Schrempf (MSc)^3^, Edith Hofer (PhD)^1,4^, Alexander Pichler (MD, PhD)^1^, Simon Fandler-Höfler (MD, PhD)^1^, Melanie Haidegger (MD)^1^, Isra Hatab (MD)^1^, Martin Heine (MD)^5^, Jan Jagiello (MD)^6^, Herbert Koller (MD)^7^, Stefan Lilek (MD)^8^, Sai Veeranki (PhD)^2^, Christian Enzinger (MD)^1^, Thomas Gattringer (MD, PhD)^1^, Markus Kneihsl (MD, PhD)^1,9^

*^1^ Department of Neurology, Medical University of Graz, Graz, Austria*

*^2^ Directorate Technology and IT, Steiermärkische Krankenanstaltengesellschaft m.b.H. (KAGes), Graz, Austria*

*^3^ Department of Internal Medicine, Division of Cardiolody, Medical University of Graz, Graz, Austria*

*^4^ Institute for Medical Informatics, Statistics and Documentation, Medical University of Graz, Graz, Austria*

*^5^ Department of Neurology, LKH Oststeiermark, Feldbach, Austria*

*^6^ Department of Neurology, LKH Murtal, Knittelfeld, Austria*

*^7^ Department of Neurology, LKH Graz II, Graz, Austria*

*^8^ Department of Neurology, LKH Hochsteiermark, Bruck/Mur, Austria*

*^9^ Division of Neuroradiology, Department of Radiology, Medical University of Graz, Graz, Austria*

**Supplemental Table 1:** Overview of delirium risk factors as presented in previous studies.

| Variables | Studies of hospitalized non-stroke patients | | | Studies of stroke patients |
| --- | --- | --- | --- | --- |
| Demographics and medical history | | | | |
| Age | Vreeswijk et al.^1^; Ormseth et al.^2^; van den Boogaard et al.^3^ | | Oldenbeuving et al.^4^; Alvarez-Perez et al.^5^; Fleischmann et al.^6^; Gustafson et al.^7^; Kostalova et al.^8^; Miu et al.^9^, Shaw et al.^10^, Pasinska et al.^11^, Zhang et al.^12^ | |
| Male sex | Ormseth et al.^2^ | | Gustafson et al.^7^, Shaw et al.^10^ | |
| Vascular risk factors | | | | |
| Arterial hypertension | Ormseth et al.^2^ | | - | |
| Hyperlipidemia | - | | Alvarez-Perez et al.^5^ | |
| Diabetes | Ormseth et al.^2^ | | Miu et al.^9^, Pasinska et al.^11^ | |
| Smoking | Ormseth et al.^2^ | | Zhang et al.^12^ | |
| Chronic alcohol consumption | Ormseth et al.^2^ | | Shaw et al.^10^, Zhang et al.^12^ | |
| Artrial fibrillation | Ormseth et al.^2^ | | Alvarez-Perez et al.^5^, Gustafson et al.^7^, Pasinska et al.^11^, Zhang et al.^12^ | |
| Laboratory parameters | | | | |
| Leucocytes | Ormseth et al.^2^, van den Boogaard et al.^3^ | | Alvarez-Perez et al.^5^, Kostalova et al.^8^, Pasinska et al.^11^ | |
| Platelets | - | | Kostalova et al.^8^ | |
| Blood urea | van den Boogaard et al.^3^ | | Oldenbeuving et al.^4^, Kostalova et al.^8^ | |
| Creatinine | Ormseth et al.^2^; van den Boogaard et al.^3^ | | Oldenbeuving et al.^4^, Kostalova et al.^8^, Pasinska et al.^11^ | |
| Blood sodium | Ormseth et al.^2^, van den Boogaard et al.^3^ | | Oldenbeuving et al.^4^, Alvarez-Perez et al.^5^, Kostalova et al.^8^ | |
| Blood potassium | Ormseth et al.^2^, van den Boogaard et al.^3^ | | Pasinska et al.^11^ | |
| Bilirubine | - | | Kostalova et al.^8^ | |
| GGT | - | | Kostalova et al.^8^ | |
| Glucose | Ormseth et al.^2^ | | Oldenbeuving et al.^4^, Pasinska et al.^11^ | |
| CRP | - | | Fleischmann et al.^6^, Pasinska et al.^11^ | |
| Medical history | | | | |
| Cognitive impairment | Vreeswijk et al.^1^; Ormseth et al.^2^ | | Oldenbeuving et al.^4^; Alvarez-Perez et al.^5^; Gustafson et al.^7^, Kostalova et al.^8^, Miu et al.^9^, Shaw et al.^10^, Pasinska et al.^11^, Zhang et al.^12^ | |
| Previous stroke | Ormseth et al.^2^ | | Alvarez-Perez et al.^5^, Miu et al.^9^ | |
| Previous delirium | Vreeswijk et al.^1^, Ormseth et al.^2^ | | Gustafson et al.^7^, Kostalova et al.^8^ | |
| Renal insufficiency | Ormseth et al.^2^ | | Miu et al.^9^ | |
| Chronic liver disease | - | | Kostalova et al.^8^, Miu et al.^9^ | |
| Psychiatric disease | Ormseth et al.^2^ | | - | |
| Previous heart disease | Ormseth et al.^2^ | | Gustafson et al.^7^, Miu et al.^9^ | |
| Malnutrition | Ormseth et al.^2^ | | - | |
| Infection | Ormseth et al.^2^; van den Boogaard et al.^3^ | | Oldenbeuving et al.^4^; Alvarez-Perez.et al.^5^; Fleischmann et al.^6^; Gustafson et al.^7^, Kostalova et al.^8^, Miu et al.^9^, Pasinska et al.^11^, Zhang et al.^12^ | |
| Visual/hearing impairment | Vreeswijk et al.^1^; Ormseth et al.^2^ | | Pasinska et al.^11^ | |
| Epilepsy | Ormseth et al.^2^ | | - | |
| Multimorbidity | Vreeswijk et al.^1^, Ormseth et al.^2^ | | Miu et al.^9^, Pasinska et al.^11^ | |
| Mobility problems | Vreeswijk et al.^1^; Ormseth et al.^2^ | | Miu et al. ^9^, Shaw et al.^10^, Pasinska et al.^11^ , Zhang et al.^12^ | |
| Medication | | | | |
| Sedative medication | Ormseth et al.^2^ | | Gustafson et al.^7^, Kostalova et al.^8^, Miu et al. ^9^ | |
| Number of medications | Vreeswijk et al.^1^; Ormseth et al.^3^ | | Kostalova et al.^8^, Shaw et al.^10^ | |
| Stroke specific factors | | | | |
| Infarct localization (anterior/posterior circulation) | - | Oldenbeuving et al.^4^; Alvarez-Perez et al.^5^; Fleischmann et al.^6^, Kostalova et al.^8^, Zhang et al.^12^ | | |
| Lacunar stroke | - | Alvarez-Perez et al.^5^, Fleischmann et al.^6^ | | |
| Infarct localization (infratentorial/supratentorial) | - | Alvarez-Perez et al.^5^, Pasinska et al.^11^ | | |
| Left/right hemispheric stroke | - | Oldenbeuving et al.^4^, Gustafson et al.^13^, Pasinska et al.^11^ | | |
| Severe stroke syndromes (based on NIHSS at admission) | - | Oldenbeuving et al.^4^, Alvarez-Perez et al.^5^, Kostalova et al.^8^, Shaw et al.^10^, Pasinska et al.^11^ | | |
| Aphasia | - | Pasinska et al.^11^, Zhang et al.^12^ | | |
| Neglect | - | Kostalova et al.^8^, Pasinska et al.^11^ | | |
| Dysphagia | Ormseth et al.^2^ | - | | |

CRP: c-reactive protein, GGT: gamma-glytamyl transferase, NIHSS: National Institutes of Health Stroke scale

**Supplemental Table 2:** Demographics, medical history and clinical/laboratory parameters of the development and the validation cohort.

| Variables | Derivation-Cohort, n=6,151 | | Validation-Cohort, n=8,324 | | |  |  |
| --- | --- | --- | --- | --- | --- | --- | --- |
| Demographics | | | | | |  |  |
| Age, median (Q1, Q3) | 76 (65, 83) | | 76 (66, 83) | | |  |  |
| Age >70, n (%) | 4092 (66.5) | | 5645 (67.8) | | |  |  |
| Male sex, n (%) | 3254 (52.9) | | 3776 (45.3) | | |  |  |
| Delir, n (%) | 398 (6.5) | | 568 (6.8) | | |  |  |
| Medical history, n (%) | | | | | |  |  |
| Atrial fibrillation | 1784 (29) | | 2513 (30.2) | | |  |  |
| Arterial hypertension | 5177 (84.2) | | 7017 (84.3) | | |  |  |
| Diabetes | 1530 (24.9) | | 2202 (26.5) | | |  |  |
| Smoking | 1136 (18.5) | | 1418 (17.0) | | |  |  |
| Chronic alcohol consumption | 633 (10.3) | | 825 (9.9) | | |  |  |
| Dementia | 457 (7.4) | | 610 (7.3) | | |  |  |
| Previous delirium | 80 (1.3) | | 106 (1.3) | | |  |  |
| Substance abuse | 519 (8.4) | | 726 (8.7) | | |  |  |
| Renal insufficiency | 910 (14.8) | | 1284 (15.4) | | |  |  |
| Chronic liver disease | 2381 (38.7) | | 3265 (39.2) | | |  |  |
| Previous heart disease | 3753 (61) | | 5326 (64) | | |  |  |
| Psychiatric disease | 1159 (18.8) | | 1732 (20.8) | | |  |  |
| Malnutrition | 93 (1.5) | | 157 (1.9) | | |  |  |
| Visual/hearing impairment | 2078 (33.8) | | 2958 (35.5) | | |  |  |
| Multimorbidity (≥2 chronic diagnosis) | 4251 (69.1) | | 5912 (71.0) | | |  |  |
| Clinical parameters | |  | |  |  | |  |
| Infection, n (%) | 2556 (41.6) | | 3505 (42.1) | | |  |  |
| Pre admission mRS, median (Q1, Q3) | 0 (0, 1) | | 0 (0,1) | | |  |  |
| NIHSS>7 at admission, n (%) | 1728 (28.1) | | 2373 (28.5) | | |  |  |
| Aphasia, n (%) | 1511 (24.6) | | 2130 (25.6) | | |  |  |
| Neglect, n (%) | 713 (11.6) | | 1034 (12.4) | | |  |  |
| Dysphagia, n (%) | 547 (8.9) | | 803 (9.6) | | |  |  |
| Stroke syndrome, n (%) | | | | | |  |  |
| Anterior circulation stroke | 3314 (53.9) | | 4595 (55.2) | | |  |  |
| Posterior circulation stroke | 1066 (17.3) | | 1419 (17.0) | | |  |  |
| Non-lacunar stroke | 4464 (72.6) | | 6117 (73.5) | | |  | |
| Left hemispheric stroke | 2798 (45.5) | | 3701 (44.5) | | |  | |
| Medication at admission, n (%) | | | | | |  | |
| Polypharmacy (≥5 drugs) | 1435 (23.3) | | 2110 (25.3) | | |  | |
| Sedative medication | 125 (2) | | 189 (2.3) | | |  | |

mRS: modified Rankin scale, NIHSS: National Institutes of Health Stroke scale

**Supplemental Table 3:** Multicollinearity assessment based on variance inflation factor for predictors of delirium.

| **Variable** | **Variance inflation factor** |
| --- | --- |
| Age (>70) | 1.334 |
| Male sex | 1.115 |
| NIHSS >7 on admission | 1.250 |
| Atrial fibrillation | 1.162 |
| Non-lacunar stroke | 1.071 |
| Dementia/cognitive impairment | 1.074 |
| Previous delirium | 1.032 |
| Chronic alcohol consumption | 1.068 |
| Visual/hearing impairment | 1.111 |
| Infection at admission | 1.133 |
| Abnormal hematocrit | 1.100 |
| Dysphagia | 1.104 |

**Supplemental Table 4:** Variables included in preexisting delirium prediction scores^1, 4^

| Oldenbeuving et al. Model 1 (points) | | | | Oldenbeuving et al. Model 2 (points) | | | Delirium risk assessment score (points) | |
| --- | --- | --- | --- | --- | --- | --- | --- | --- |
| Age | | | (0-6) | Age | | (0-6) | Age | (1) |
| Stroke severity (NIHSS) | | | (0-13) | Stroke severity (NIHSS) | | (0-10) | Chronic alcohol consumption | (3) |
| Stroke subtype (PACI/TACI; POCI; ICH) | | (5; 2; -2) | | Stroke subtype (PACI/TACI; POCI; ICH) | (3; 1; -1) | | History of delirium | (1) |
| Infection | | | (6) | Infection | | (4) | Pre-existing mobility problems | (2) |
| Right hemispheric stroke | | | (3) |  | |  | Cognitive impairment | (3) |
| Cognitive impairment | | | (6) |  | |  | Acute admission | (3) |
|  | | |  |  | | | Visual/hearing impairment | (1) |
|  | | |  |  | | | Polypharmacy (≥5 medications) | (1) |
|  | -2-39 | | | -1-13 | | | 0-5 | |

c

PACI/TACI: partial/total anterior circulation infarct, POCI: posterior circulation infarct, ICH: intracerebral hemorrhage

NIHSS: National Institutes of Health Stroke scale

**Supplemental Table 5**: Additional laboratory parameters on admission of the derivation stroke, categorized by the presence of delirium.

| Variables | All ischemic stroke patients (n=6,151) | Patients with delirium (n=398) | Patients without delirium (N=5,753) | OR (95% CI) | p-value | |
| --- | --- | --- | --- | --- | --- | --- |
| Laboratory Variables | | | | | | |
| Platelets <140/>440 *10^9/L | 482 (7.8) | 48 (12.1) | 434 (7.5) | 1.68 (1.22-2.3) | 0.003 | |
| Blood urea >45mg/dL | 1496 (24.3) | 108 (27.1) | 1388 (24.1) | 1.17 (0.93-1.47) | 0.184 | |
| MCV <80/>98 fL | 588 (9.6) | 40 (10.1) | 548 (9.5) | 1.06 (0.76-1.49) | 0.724 | |
| Sodium <135/>145 mmol/L | 630 (10.2) | 40 (10.1) | 590 (10.3) | 0.98 (0.7 - 1.37) | 1 | |
| Potassium <3.5/>5 mmol/L | 818 (13.3) | 67 (16.8) | 751 (13.1) | 1.35 (1.02-1.77) | 0.039 | |
| Bilirubine >1.2 mg/dL | 322 (5.2) | 39 (9.8) | 383 (6.7) | 1.52 (1.08-2.15) | 0.023 | |
| AST >35 U/L | 895 (14.6) | 75 (18.8) | 810 (14.1) | 1.42 (1.09-1.84) | 0.012 | |
| ALT >35 U/L | 415 (6.7) | 32 (8) | 383 (6.7) | 1.23 (0.84-1.79) | 0.300 | |
| Lipase >60 U/L | 28 (0.5) | 3 (0.8) | 25 (0.4) | 1.74 (0.52-5.79) | 0.424 |  |
| Glucose <70/>100 mg/dL | 3779 (61.4) | 262 (65.8) | 3517 (61.1) | 1.23 (0.99-1.52) | 0.070 |  |

ALT: Alanine aminotransferase AST: Aspartate aminotransferase, CI: confidence interval; CRP: c-reactive protein; MCV: mean corpuscular volume, NIHSS: National Institutes of Health Stroke scale; OR: odds ratio

**Supplemental Table 6:** Diagnostic test evaluation of delirium risk scores at different cutoffs in acute ischemic stroke patients for their sensitivity, specificity, positive likelihood ratio and accuracy for delirium development.^1, 4^

|  | Low-Risk cutoff (≤5% risk) | | | | | High-Risk cutoff (≥20% risk) | | | | |
| --- | --- | --- | --- | --- | --- | --- | --- | --- | --- | --- |
| Variable/ cutoffs | Sensitivity (%) | Specificity (%) | | Likelihood ratio (+, %) | Accuracy | Sensitivity (%) | Specificity (%) | | Likelihood ratio (+, %) | Accuracy |
| **Delirium risk score (admission)** | |  |  | |  |  |  |  | |  |
| PREDELIS score (Derivation cohort, ≤5 points) | 63.4 | 70.1 | 1.91 | | 62.4 | 22.1 | 94.8 | 4.25 | | 90.1 |
| PREDELIS score (Validation cohort, ≤5 points) | 61.8 | 70.0 | 1.83 | | 62.4 | 23.2 | 94.4 | 4.22 | | 89.6 |
| Oldenbeuving et al. (Model 1; ≤17 points) | 49.8 | 74.1 | 1.48 | | 51.4 | - | - | - | | - |
| Oldenbeuving et al. (Model 2; ≤12 points) | 49.5 | 73.9 | 1.46 | | 51.1 | - | - | - | | - |
| DRAS (≤1 point) | 48.1 | 71.8 | | 1.38 | 49.7 | - | - | | - | - |

DRAS: Delirium risk assessment score; PREDELIS: Predicting delirium in acute ischemic stroke

**Supplemental Figure 1:** Diagnostic criteria of delirium according to international classification of diseases (ICD)-10.^14^


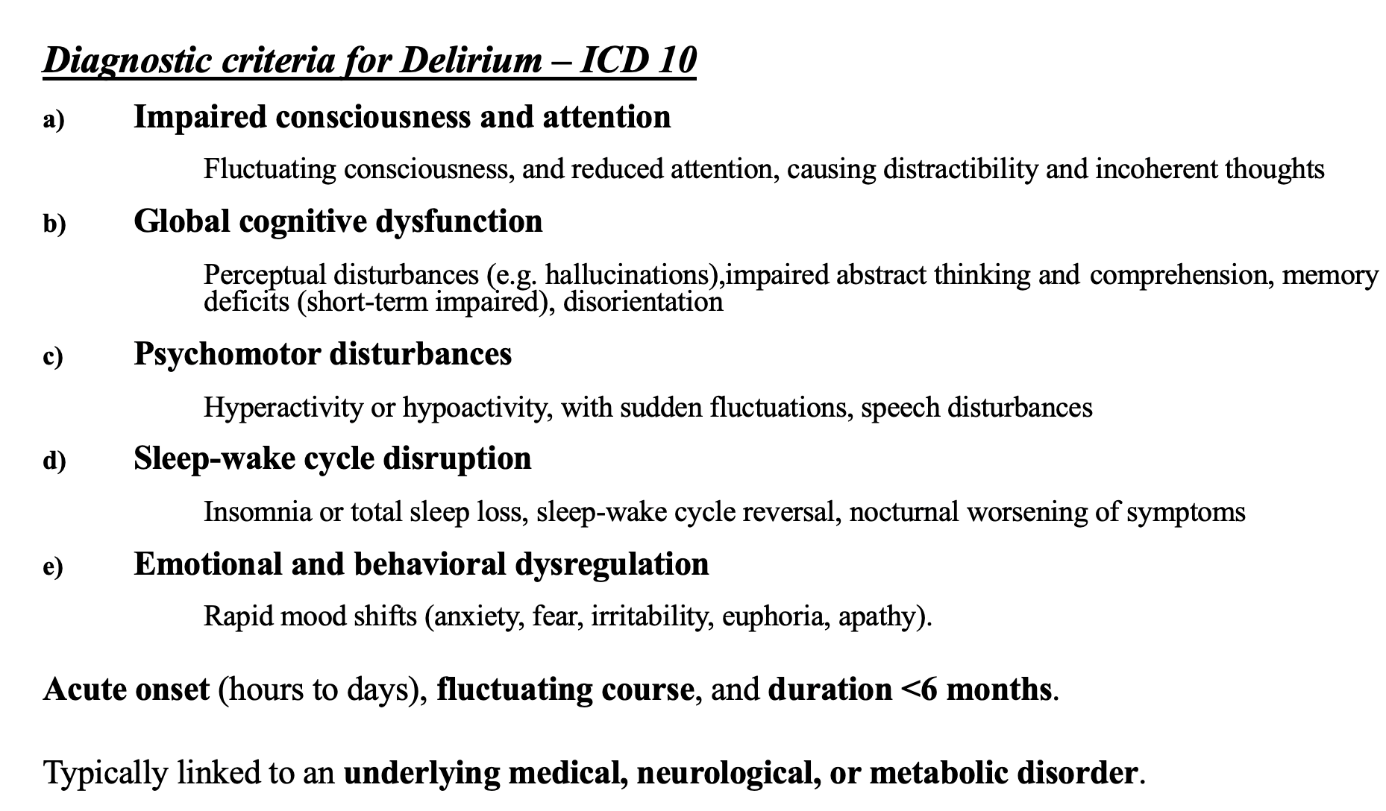


**Supplemental Figure 2:** Flowchart of patients who met inclusion/exclusion criteria in the study population.

***
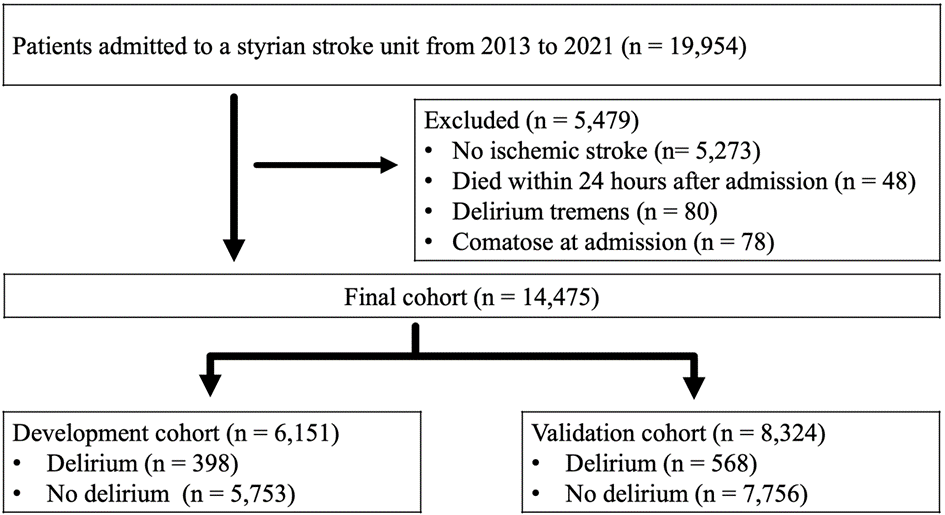
***


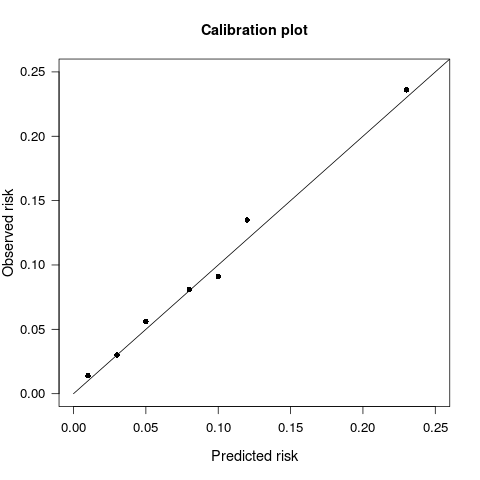
**Supplemental Figure 3:** Calibration plot of predicted versus observed probabilities of delirium risk according to the PREDELIS score (derivation versus validation cohort).*

*Data point 1 summarizes PREDELIS risk score points of 0-2. Data point 2 represents the risk for scores of 3-4 points, as the individual risks are very similar. Each subsequent point corresponds to one additional point in the PREDELIS risk score up to ≥9 score points.

*Calibration Plot*

# Supplemental References

1. Vreeswijk, R.; Kalisvaart, I.; Maier, A. B.; Kalisvaart, K. J. Development and validation of the delirium risk assessment score (DRAS), *Eur Geriatr Med*. **2020, *11,*** 307-314.

2. Ormseth, C. H.; LaHue, S. C.; Oldham, M. A.; Josephson, S. A.; Whitaker, E.; Douglas, V. C. Predisposing and Precipitating Factors Associated With Delirium: A Systematic Review, *JAMA Netw Open*. **2023, *6,*** e2249950.

3. van den Boogaard, M.; Pickkers, P.; Slooter, A. J., et al. Development and validation of PRE-DELIRIC (PREdiction of DELIRium in ICu patients) delirium prediction model for intensive care patients: observational multicentre study, *BMJ*. **2012, *344,*** e420.

4. Oldenbeuving, A. W.; de Kort, P. L.; van Eck van der Sluijs, J. F.; Kappelle, L. J.; Roks, G. An early prediction of delirium in the acute phase after stroke, *J Neurol Neurosurg Psychiatry*. **2014, *85,*** 431-434.

5. Alvarez-Perez, F. J.; Paiva, F. Prevalence and Risk Factors for Delirium in Acute Stroke Patients. A Retrospective 5-Years Clinical Series, *J Stroke Cerebrovasc Dis*. **2017, *26,*** 567-573.

6. Fleischmann, R.; Andrasch, T.; Warwas, S., et al. Predictors of post-stroke delirium incidence and duration: Results of a prospective observational study using high-frequency delirium screening, *Int J Stroke*. **2023, *18,*** 278-284.

7. Gustafson, Y. O., T.; Eriksson, S.; Asplund, K.; Bucht, G. Acute Confusional States (Delirium) in Stroke Patients, *Cerebrovasc Dis*. **1991, *1:257-264***.

8. Kostalova, M.; Bednarik, J.; Mitasova, A., et al. Towards a predictive model for post-stroke delirium, *Brain Inj*. **2012, *26,*** 962-971.

9. Miu, D. K.; Chan, C. W.; Kok, C. Delirium among elderly patients admitted to a post-acute care facility and 3-months outcome, *Geriatr Gerontol Int*. **2016, *16,*** 586-592.

10. Shaw, R. C.; Walker, G.; Elliott, E.; Quinn, T. J. Occurrence Rate of Delirium in Acute Stroke Settings: Systematic Review and Meta-Analysis, *Stroke*. **2019, *50,*** 3028-3036.

11. Pasinska, P., Kowalska, K.; Klimiec, E.; Szyper‐Maciejowska, A.; Wilk, A.; Klimkowicz‐Mrowiec, A. Frequency and predictors of post‐stroke delirium in PRospective Observational POLIsh Study (PROPOLIS), *Journal of Neurology*. **2018, *265(4):863-870***.

12. Zhang, G. B.; Li, H. Y.; Yu, W. J., et al. Occurrence and risk factors for post-stroke delirium: A systematic review and meta-analysis, *Asian J Psychiatr*. **2024, *99,*** 104132.

13. Y. Gustafson, T. O., K. Asplund, and E. Hagg Acute confusional state (delirium) soon after stroke is associated with

hypercortisolism, *Cerebrovascular Diseases*. **1993, *3,*** 33-38.

14. (WHO), W. H. O. International Statistical Classification of Diseases and Health Related Problems—Tenth Revision.). Geneva: World Health Organization, 1993.
